# Giant Left Atrial Myxoma in a Nonagenarian

**DOI:** 10.1111/jgs.12059

**Published:** 2013-01-11

**Authors:** Yoshihisa Matsumura, Yasuteru Nakashima, Tatsuya Noguchi, Yuichi Baba, Michiko Wada, Kayo Hayashi, Toru Kubo, Naohito Yamasaki, Takashi Furuno, Hiroaki Kitaoka, Kazumasa Orihashi, Tetsuro Sugiura, Yoshinori Doi

**Affiliations:** 1 Department of Medicine and Geriatrics, Kochi Medical School, Kochi UniversityKochi, Japan; 2 Department of Laboratory Medicine, Kochi Medical School, Kochi UniversityKochi, Japan; 3 Department of Surgery II, Kochi Medical School, Kochi UniversityKochi, Japan

*To the Editor:* An independent 90-year-old man presented with progressive shortness of breath. A left atrial mass had been accidentally found on chest computed tomography at another hospital 2 years earlier. He had declined further investigation and treatment for the mass. He had been asymptomatic until 3 months earlier. He had undergone a unilateral nephrectomy for renal cell carcinoma 17 years before and had been free of recurrence after surgery. His family history was unremarkable. On physical examination, his pulse rate was 63 beats per minute, and blood pressure was 132/62 mmHg. He had bilateral leg edema and a distended jugular vein. An accentuated first heart sound without tumor plop or diastolic murmur was heard. Breath sounds were diminished. Electrocardiogram was unremarkable. Chest X-ray showed bilateral pleural effusion. Transthoracic and transesophageal echocardiography revealed a mobile and pedunculated mass in the left atrium attached to the interatrial septum (Figure 1A). The mass prolapsed into the left ventricle across the mitral valve, resulting in mitral valve obstruction and pulmonary hypertension. Tricuspid regurgitation was mild, with a pressure gradient of 53 mmHg. The inferior vena cava was 18 mm in diameter, with low respiratory change. The mitral valve appeared structurally normal, and mitral regurgitation was trivial. The left ventricle was normal in size and function. He was open to the prospect of surgical removal of the mass and accepted after obtaining the details of the surgery and discussing matters with us and his family. He underwent surgical resection of the mass (Figure 1B). Histologic examination confirmed a diagnosis of myxoma. Postoperatively, pressure gradient calculated from tricuspid regurgitation decreased to 27 mmHg. He recovered without major complication and was discharged.

**Figure 1:**
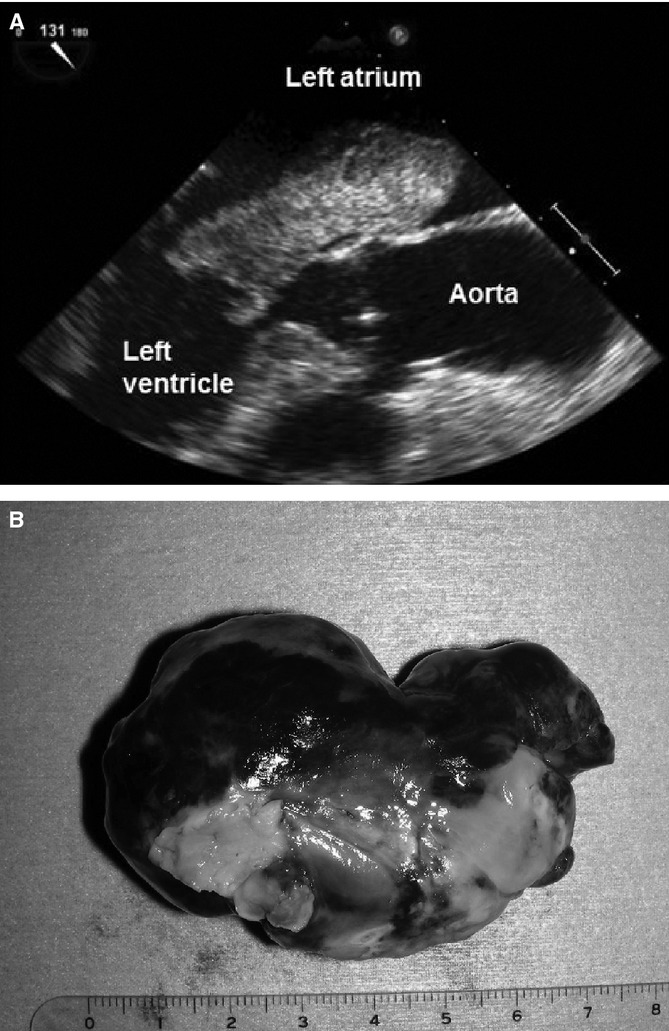
(A) Transesophageal echocardiogram showing an atrial mass prolapsing into the left ventricle. (B) The excised myxoma (3.5 × 4.5 × 7 cm).

## Discussion

Left atrial myxoma is histologically benign and is the most common primary tumor of the heart.^1^ It is particularly frequent between the third and sixth decades of life. Atrial myxoma in older people is considered to be unusual, but with the advent of newer imaging modalities and longer life expectancy, the incidence of myxoma in older people has increased.^2^,^3^ In a series of 100 individuals with atrial myxoma, 19% were aged 70 and older.^2^ A recent review also reported 63 individuals with cardiac myxoma aged 68 to 88.^3^ Cardiac myxomas in older people were surgically resected with mortality of 1.7%.^3^ Most of their presenting symptoms resolved after surgical removal. To the best of the authors’ knowledge, the current patient is the oldest person with atrial myxoma who has undergone surgical resection.^3^,^4^ Atrial myxoma is among the great mimickers of clinical medicine.^2011^ It should be put on a list of differential diagnoses for heart failure even in older people. Surgical resection could lead to resolution of symptoms and avoidance of devastating complications.

## 

**Conflict of Interest**: The editor in chief has reviewed the conflict of interest checklist provided by the authors and has determined that the authors have no financial or any other kind of personal conflicts with this paper.

**Author Contributions**: YM: Imaging, management of the patient, writing the report. MW, KH: Imaging. YN, YB, TK, NY: Management of the patient. TF, HK, KO, YD: Management of the patient, reviewing the text. TS: Imaging, reviewing the text.

**Sponsor's Role**: None.
